# Distribution and association of hs-CRP with cardiovascular risk variables of metabolic syndrome in adolescent learners

**DOI:** 10.4102/ajlm.v1i1.10

**Published:** 2012-06-04

**Authors:** Megan A. Rensburg, Tandi Matsha, Mariza Hoffmann, Mogamat S. Hassan, Rajiv T. Erasmus

**Affiliations:** 1 Division of Chemical Pathology, National Health Laboratory Service, Tygerberg Hospital, Stellenbosch University, South Africa; 2 Faculty of Health and Wellness Sciences, Cape Peninsula University of Technology, South Africa

## Abstract

**Objective:**

Metabolic syndrome (MetS) and its associated cardiovascular risk are on the increase in children. High-sensitivity C-reactive protein (hs-CRP) has emerged as a useful marker for inflammation associated with atherosclerosis and cardiovascular disease. Our aim was to determine the distribution of hs-CRP in an effort to identify the MetS variable that is critical in modulating plasma CRP levels in a population of South African adolescents.

**Design:**

A cross-sectional analytical study design was used for this investigation, where the dependent and independent variables were measured simultaneously.

**Methods:**

Anthropometric variables, blood pressure, fasting blood glucose and lipids were performed on 324 consenting learners aged 15–18 years from three different ethnic groups (Black, White and Coloured). The National Cholesterol Education Program Adult Treatment Panel III (NCEP ATP III) for ages 15–18 year olds was used to define MetS.

**Results:**

The prevalence of MetS and obesity was 3.7% and 7.1%, respectively. The hs-CRP levels were significantly higher in subjects with a waist-circumference greater than the 90th percentile (*p* < 0.01) and in obese learners with MetS, but was lower in adolescents with normal weight and MetS. Median hs-CRP levels increased with an increasing number of metabolic abnormalities and exceeded 3 mg/L in 19% of adolescents. Gender and ethnic differences were observed.

**Conclusion:**

Our findings suggest that obesity and waist circumference appear to be major mediators of hs-CRP levels in South African adolescents.

## Introduction

Globally, obesity and its accompanying metabolic complications are on the increase. Childhood obesity has reached epidemic proportions and is one of the most serious public health problems facing the developed and, increasingly, the developing world.^1^ Metabolic syndrome (MetS), a constellation of interrelated conditions of insulin resistance, hyperlipidaemia, hypertension and obesity, is one of the recognised complications or associations of obesity. A pro-inflammatory and pro-thrombotic state contributing to endothelial dysfunction is a common feature of those with MetS.^2^ It is well recognised that inflammation plays a key role in the pathophysiology of atherosclerosis and cardiovascular disease (CVD).^3^

Markers of endothelial dysfunction and inflammation include tumour necrosis factor-alpha (TNF-α), the interleukins (IL) and C-reactive protein (CRP). TNF-α is a cytokine secreted from endothelial and smooth muscle cells, macrophages and fat cells. It enhances monocyte recruitment into developing atherosclerotic lesions and therefore links obesity with atherosclerosis.^4^ IL-6 is the major pro-coagulant cytokine. It increases plasma levels of plasminogen activator inhibitor type 1, fibrinogen and CRP. Lifestyle modifications, including a Mediterranean-style diet and weight loss, reduce serum concentrations of IL-6, IL-7, and IL-18.^5^ Patients with coronary heart disease have markedly elevated levels of IL-1, especially those with unstable disease.^6^

Pro-inflammatory cytokines, including IL1-β, IL-6 and TNF-α, are released from macrophages within the vessel wall during an inflammatory response.^4^ These cytokines mediate distant inflammatory effects, including activation of hepatic genes encoding acute-phase reactants, CRP, fibrinogen and serum amyloid A. CRP induces synthesis of other cytokines, cellular adhesion molecules (CAMs) and tissue factor in monocytes and endothelial cells.^4,7^ Tissue factor activation provides the link between inflammation and coagulation by activating the coagulation cascade.

CRP was traditionally measured in patients to assess and monitor systemic inflammation during an acute infection. Values exceeding 10 mg/L are accepted as evidence of probable infection and/or sepsis. The need for detection very low levels (< 3 mg/L) only became necessary when CRP was discovered to be a useful marker of endothelial dysfunction and future CVD risk. High-sensitivity C-reactive protein (hs-CRP) has emerged as a useful biomarker for vascular inflammation associated with atherosclerosis and it may directly promote atherosclerotic processes.^8,9^ As such, elevated hs-CRP levels, as a response to the increase in the secretion of cytokines of adipose origin, have been used as a marker of cardiovascular risk and diabetes in adults.^10^ Various hs-CRP methods have been introduced, fulfilling the need of measuring CRP levels at these low levels. In apparently healthy adults, hs-CRP levels of more than 3 mg/L are associated with a high risk of future CVD.

Many studies have examined the clustering of metabolic abnormalities, but the metabolic phenotype continues to be less well understood in children in terms of criteria, prevalence and clinical implications.^11^ Studies have reported high values of hs-CRP amongst obese children and adolescents, indicating a certain level of inflammation.^3,12^ Childhood CRP values have also been shown to predict adult CRP levels independently of other metabolic risk factors.^13^ The US National Health and Nutrition Examination Survey (USNHANES) reported that adolescents with more metabolic abnormalities had higher CRP levels.^11^

The few studies conducted in developing countries have shown a relatively high prevalence of MetS that is paralleled by the increasing obesity in children and adolescents.^14^ A recent study on the prevalence of MetS amongst South African children reported an overall rate of 6.5% using the National Cholesterol Education Program expert panel on detection, evaluation and treatment of high blood cholesterol – Adult Treatment Panel III (NCEP ATP III) definition.^15^ In South Africa, morbidity and mortality related to non-communicable disease remain prominent, despite the sustained increase in deaths from chronic infections such as HIV and AIDS and tuberculosis.^16,17^ This is particularly important when providing interventions that target CVD and their risk factors, as these chronic conditions place a huge financial and human resource burden on South Africa’s health budget. The measurement of hs-CRP concentrations may thus provide an indication of future risk of CVD, which may lead to earlier intervention.

Therefore, the aim of this study was to determine the distribution of hs-CRP in a population of South African adolescent learners and to determine its association with cardiovascular risk factor variables of MetS, in an effort to identify the variable that is critical in modulating plasma hs-CRP levels in this population.

## Materials and methods

### Subjects

Our study population formed part of a larger study involving 1683 learners aged 8–18 years, recruited through a proportionally stratified multistage random sampling technique from government-funded secondary schools in the Western Cape Province of South Africa. The inclusion criteria were, (1) male or female learners aged 15 years – 18 years, (2) fasted overnight (≥ 10 h) and (3) no current or recent history of infectious disease (on self-report). Participants were excluded if the measured hs-CRP values were > 10 mg/L, or if there were any missing data. Recent and/or current infectious disease was excluded by self-report and brief medical examination on the day of sampling.

### Measurement of blood pressure, glucose and lipids

Finger prick blood performed by trained nursing staff was used for the estimation of fasting blood glucose (FBG) and lipid levels using the Accutrend GCT glucometer and CardioChek^TM^ PA analyser (Polymer Technology Systems, Inc., Indianapolis, USA), respectively. The glucometer coefficient of variation is stated as < 3% and accuracy is approximately 5%, when compared to a hexokinase method. The Cardiochek^TM^ analyser showed reasonable compliance with NCEP goals for coefficients of variation and bias measurements.^18^ Blood pressure measurements were performed using a semi-automatic digital blood pressure monitor (Rossmax Int. Ltd, Taipei, Taiwan) according to World Health Organization guidelines.^19^ The NCEP ATP III for ages 15–18 years were used to define MetS^20^ (Table 1). Individuals were classified as overweight or obese, according to age and gender, as described by Cole et al.^21^

**TABLE 1:** Criteria for the metabolic syndrome are listed below and can be any three of more.

Criteria list	Components
Central obesity^†^	> 90th percentile, age and gender specific
FBG	≥ 5.6 mmol/L (100 mg/dL) or known type 2 diabetes
Hypertension	≥ 90th percentile for age, sex and height
TG	≥ 1.24 mmol/L (110 mg/dL)
HDL-C	≤ 1.04 mmol/L (40 mg/dL)

*Source*: Cook S, Weitzman M, Auinger P, Nguyen M, Dietz WH. Prevalence of a metabolic syndrome phenotype in adolescents. Arch Pediatr Adolesc Med. 2003;157:821–827.

FBG, fasting blood glucose; TG, triglycerides; HDL-C, high density lipoprotein cholesterol.

† Waist circumference.

### Measurement of hs-CRP

Different platforms exist to measure hs-CRP, including turbidimetric, nephelometric and immunometric assays (two-site chemiluminescent). These different methodologies compare well in precision and comparative studies.^22^ Venous blood was collected and the serum was separated and frozen at -20 °C before batch analysis. The hs-CRP was measured by an hs-CRP assay, based on the highly sensitive Near Infrared Particle Immunoassay rate methodology (Immage^®^ Immunochemistry System, Beckman Coulter Inc., Brea, USA), with an analytical sensitivity of 0.2 mg/L and a functional sensitivity of 0.11 mg/L (measuring range 0.2 mg/L – 1440 mg/L). Participants with hs-CRP concentrations < 0.2 mg/L had a value assigned to them of 0.2 mg/L.

### Statistical analysis

The STATISTICA 8 software package (StratSoft Inc., Tulsa, USA) was used to perform statistical analyses. Descriptive data are represented as medians because hs-CRP has a non-Gaussian distribution and non-parametric statistics have been applied. Other variables, such as glucose, triglycerides and cholesterol, are presented as means, as they follow a normal distribution. For categorical data, group comparisons were performed using the Mann Whitney U-test. A 5% significance level (*p* < 0.05) was used for determining significant differences.

## Ethical considerations

The study was approved by the Faculty of Health Science Ethics Committee at the University of Stellenbosch and consent to conduct the study was obtained from the Western Cape Education Department. Ethical clearance was given for the collection of venous blood samples from the participants older than 12 years. Finally, a parent or guardian gave consent for the adolescents’ participation in this study and the participants themselves assented.

## Results

Three-hundred-and-twenty-nine learners who satisfied the inclusion criteria were recruited for the study. Five were then excluded from this initial study population as a result of hs-CRP levels > 10 mg/L, leaving 324 learners for this study. Of this total, 186 (57.4%) were female, whilst 138 (42%) were male. The final study population comprised 136 (42%) Black learners, 62 (19%) White learners and 126 (39%) Coloured learners.

The distribution of hs-CRP was skewed, with higher median hs-CRP concentrations in female learners than male learners (1.77 mg/L vs 1.39 mg/L, respectively; *p* = 0.15). Significant racial differences were observed with higher median hs-CRP levels in non-White learners (White – 1.02 mg/L; Black – 1.74 mg/L; Coloured – 1.76 mg/L). The hs-CRP levels correlated positively with the learners’ body mass index (BMI) and waist circumference, but not with high density lipoprotein cholesterol (HDL-C) or triglycerides.

The prevalence of MetS and obesity was 3.7% and 7.1%, respectively. Obesity was higher in all female learners, whilst Coloured learners (17%) had a nearly three times higher rate of obesity than White learners (5%) or Black learners (4%).

**FIGURE 1:**
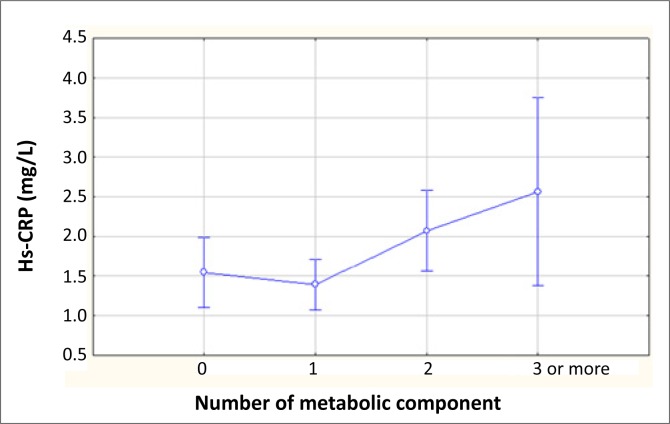
Association between the number of metabolic components and high-sensitivity C-reactive protein (Hs-CRP).

The median hs-CRP levels in the MetS group were 2.56 mg/L, compared to 1.67 mg/L in those without MetS. The hs-CRP values correlated positively with an increase in the number of MetS components present (Figure 1). Four (33%) subjects with MetS had a normal weight and their median hs-CRP levels were significantly lower than the obese group (Table 2). Because low-grade systemic inflammation may underlie, at least in part, the clustering of metabolic risk factors, we examined the relationship between hs-CRP concentrations and components of the MetS. There was no association between the individual components, blood pressure, triglycerides, fasting blood glucose and hs-CRP noted. More than 60% of the participants had HDL values below the cut-off of 1.04 mmol/L, with a mean value 0.97 mmol/L.

**TABLE 2:** Metabolic syndrome, body mass index (BMI) and high-sensitivity C-reactive protein (hs-CRP) levels amongst the study participants (*N* = 324).

BMI	*N*	Hs-CRP (mg/L)	Subjects (> 3 components)	
			Number	Median hs-CRP (mg/L)
Normal	251	1.28	4	0.65
Overweight	50	2.12	1	0.60
Obese	23	4.00	7	3.94
**Total**	**324**	**1.61**	**12**	**–**

There was a significant difference (*p* < 0.01) noted in the CRP concentrations between those with a normal waist circumference, compared to those with a waist circumference greater than the 90th percentile (1.39 mg/L vs 3.22 mg/L, respectively).

The hs-CRP levels of > 3 mg/L were seen in 18.5% (*n* = 60) of learners. Table 3 compares baseline demographic data and laboratory values for children with CRP levels of ≥ 3 mg/L and those with CRP levels of < 3 mg/L. These subjects were likely to have higher BMIs, with a higher percentage of obese subjects. However, 13% (*n* = 33) of children with levels > 3 mg/L had normal BMI.

**TABLE 3:** Baseline demographic data and laboratory results of the study participants (*N* = 324).

Category	Sub-category	hs-CRP < 3 mg/L	hs-CRP ≥ 3 mg/L	*p*-Value
Gender	Male	117 (85%)	21 (15%)	–
	Female	147 (79%)	39 (21%)	–
Race	Coloured			
	Black	99 (79%)	27 (21%)	–
	White	111 (82%)	25 (18%)	–
		54 (87%)	8 (13%)	–
Body mass index	Normal	218 (87%)	33 (13%)	–
	Overweight	37 (74%)	13 (26%)	–
	Obese	9 (39%)	14 (61%)	–
Fasting blood glucose (mmol/L)	Mean	3.86	4.25	< 0.01
HDL (mmol/L)	Mean	0.97	0.99	0.68
Triglycerides (mmol/L)	Mean	0.82	0.77	0.39
Waist circumference	< 90th percentile	244 (85%)	42 (15%)	< 0.01
> 90th percentile	20 (53%)	18 (47%)	

hs-CRP, high-sensitivity C-reactive protein; HDL, high density lipoprotein.

## Discussion

Inflammation is an important pathogenic factor in the development of atherosclerosis and coronary heart disease, especially in those with diabetes, obesity and MetS. An important asset of our study is that it investigated the prevalence of MetS in adolescents from three different racial groups in South Africa. MetS, as defined by the modified NCEP ATP III classification, was observed in 3.7% of all children, with hs-CRP values nearly three times higher in children with MetS compared to those without it.^20^ Previous studies have reported that hs-CRP concentrations correlate with individual components of the MetS.^11^ We observed a positive linear trend in hs-CRP levels, with an increasing number of features of MetS. We also observed no association between the individual components: blood pressure, triglycerides, fasting blood glucose and hs-CRP. Of the metabolic variables studied, adiposity, as measured by BMI and waist circumference, was the major predictor of hs-CRP (similar to results obtained from international studies^3,11^), suggesting obesity as a mediator of excess CRP seen in the MetS. MetS has been associated with higher CRP levels even in the absence of obesity,^3,11,23^ but this was not observed in our study. Considerably lower hs-CRP levels were observed in children with MetS but with normal weight (also termed metabolically obese, normal-weight children), with the median hs-CRP in this group being 0.65 mg/L, compared to 3.9 mg/L in the obese group. Previous studies have suggested that metabolically obese but normal-weight individuals are also at an increased risk of developing type 2 diabetes and CVD^24^; however, this needs to be explored further in our setting in the light of these observations.

This community-based study has demonstrated that hs-CRP levels in South African children vary by race and sex and relate adversely to risk variables of MetS. The hs-CRP values ranged from 0.2 mg/L to 9.9 mg/L, with higher median hs-CRP concentrations in female learners (1.77 mg/L vs 1.39 mg/L). Similar observations have been made by Lambert et al.^23^ in 16-year-old children from Quebec, Canada, as well as in the 1999–2000 US NHANES^11^ and the Bogalusa studies^3^. However, in the Taipei Children Heart Study of CRP concentrations no gender differences were observed.^25^ Sex differences have been attributed to the effect of estrogens, which have been implicated in the transcriptional control and clearance of hs-CRP.^26^

As observed in adults,^27^ we found that there were also ethnic differences, with Black and Coloured children having higher hs-CRP levels. Ethnic differences in body fat patterning are thought to account for some of the observed differences.^28^ In the Bogalusa study, hs-CRP levels showed racial differences and related to measures of obesity.^3^ For a given waist circumference, Black boys and girls from the USA were found to have higher abdominal subcutaneous adipose tissue and lower visceral adipose tissue than their White peers.^28^ Similar observations have been reported in South African children, in whom relatively more subcutaneous fat and greater waist circumference have been observed in stunted girls, which may predispose them to obesity in adulthood.^29^ In the Strong Without Anorexia Nervosa (SWAN) study, which examined 3154 women from different ethnic groups, Afro-American ethnicity was associated with higher hs-CRP levels after controlling for BMI, age and other risk factors, suggesting that genetic variants of hs-CRP and dietary and lifestyle factors could also be contributory factors.^27,30^ The implications of these elevated hs-CRP levels need further study, as South African Black people generally have a better cardiovascular risk profile than other ethnic groups.

Obesity rates were comparable to those observed in many developed countries and obese learners in our study had a significantly higher median hs-CRP (4.00 ± 0.58 mg/L) compared to their normal-weight counterparts (1.28 ± 0.12 mg/L). Because fat distribution appears to have a more important influence on cardiovascular risk factors in young subjects than overall adiposity, abdominal obesity has been identified as the main determinant of several metabolic disorders.^31,32^ The relationship of waist circumference as a measure of abdominal obesity appears to be explained by its strong association with visceral adipose tissue, therefore explaining the strong association with inflammatory biomarkers such as hs-CRP.^33^ We compared the hs-CRP concentration of those with a normal waist circumference to those individuals with a waist circumference greater than the 90th percentile and, as expected, found a significant difference (*p* < 0.01). This finding is consistent with international results and highlights that an increase in abdominal obesity mirrors visceral adiposity and increased inflammation.^33^ Waist circumference, particularly in young South African women, has been shown to be a better predictor of insulin resistance than visceral adipose tissue.^34^

Whilst these results re-affirm that high hs-CRP values are most likely caused by increased fat deposition, we also observed that more than half of the children with hs-CRP > 3 mg/L were of normal weight, suggesting that a sizable number of apparently healthy children may be at increased cardiovascular risk. Despite these observations in this and other studies, it is not recommended for hs-CRP to be a suitable target for therapeutic intervention as no causal association between hs-CRP and MetS phenotypes has been reported.^35^ One platform to measure CRP, regardless of the clinical context, is ideal in the clinical laboratory as it could lead to decreased cost and turn-around-time of analysis, as most laboratories have dedicated instruments for measurement of hs-CRP and batch-analysis is usually performed.

There are several limitations to our findings. The primary limitation is the use of a single hs-CRP measurement. In adults, repeat hs-CRP measurements are recommended, as concentrations are affected by factors such as recent infection and inflammatory conditions. The measurement of other inflammatory biomarkers of endothelial dysfunction could have added more value to the study. Other tests that could have excluded active infection, for example cytokines or white blood cell count, were not conducted. A second limitation is that this was not a national study and excluded learners from private schools. Another limitation is that the study outcomes depended on the definition of MetS. Insulin resistance, the key component of the MetS, was not determined. Furthermore, direct measurement of body fat mass and distribution was not assessed and the study was observational and cross-sectional in nature, making it difficult to differentiate between cause and effect, and associations.

In summary, we have found that hs-CRP is increased in South African adolescents with metabolic abnormalities and the MetS phenotype. Markers of adiposity, such as BMI and waist circumference, appear to be strong mediators of hs-CRP and many learners had hs-CRP levels greater than 3 mg/L, a value that has been used in adults to indicate high CVD risk. In our setting, hs-CRP may be useful marker to assess early atherosclerosis and cardiovascular risk, particularly in those children with normal BMI.
